# Responses of plant diversity to precipitation change are strongest at local spatial scales and in drylands

**DOI:** 10.1038/s41467-021-22766-0

**Published:** 2021-05-03

**Authors:** Lotte Korell, Harald Auge, Jonathan M. Chase, W. Stanley Harpole, Tiffany M. Knight

**Affiliations:** 1 Department of Community Ecology, Helmholtz Centre for Environmental Research – UFZ, Halle (Saale), Germany; 2 Institute of Biology, Martin Luther University Halle-Wittenberg, Halle (Saale), Germany; 3 German Centre for Integrative Biodiversity Research (iDiv), Halle-Jena-Leipzig, Leipzig, Germany; 4 Department of Computer Science, Martin Luther University Halle-Wittenberg, Halle (Saale), Germany; 5 Department of Physiological Diversity, Helmholtz Centre for Environmental Research – UFZ, Leipzig, Germany

**Keywords:** Biodiversity, Climate-change ecology

## Abstract

Mitigating and adapting to climate change requires an understanding of the magnitude and nature by which climate change will influence the diversity of plants across the world’s ecosystems. Experiments can causally link precipitation change to plant diversity change, however, these experiments vary in their methods and in the diversity metrics reported, making synthesis elusive. Here, we explicitly account for a number of potentially confounding variables, including spatial grain, treatment magnitude and direction and background climatic conditions, to synthesize data across 72 precipitation manipulation experiments. We find that the effects of treatments with higher magnitude of precipitation manipulation on plant diversity are strongest at the smallest spatial scale, and in drier environments. Our synthesis emphasizes that quantifying differential responses of ecosystems requires explicit consideration of spatial grain and the magnitude of experimental manipulation. Given that diversity provides essential ecosystem services, especially in dry and semi-dry areas, our finding that these dry ecosystems are particular sensitive to projected changes in precipitation has important implications for their conservation and management.

## Abstract (web summary)

The responses of terrestrial ecosystems to changes in precipitation patterns are highly context-dependent. Here the authors perform a quantitative synthesis of field rainfall manipulation experiments, showing stronger effects of precipitation on plant diversity at small spatial scales and in arid biomes.

## Introduction

Human-caused climate change has dramatically altered temperature and precipitation distribution across the planet. While there is certainly variation in temperature changes, precipitation changes are even more variable, with both positive and negative changes in different parts of the world^1^. These changes in precipitation will, in turn, influence the structure and functioning of the altered ecosystems and the biodiversity therein^2^. While model-based projections for how ecosystems will change in the face of changing precipitation regimes are valuable^3^, experimental manipulations of climate change represent the prima facie evidence for predicting the structure and function of future ecosystems. While there have been many dozens of precipitation experiments across the world, synthesis has been difficult^4–10^. For example, Yue and colleagues^10^ conducted a meta-analysis of precipitation addition experiments, and found no overall effect of the treatment on biodiversity. Furthermore, Komatsu and colleagues^7^ synthesized data from global change experiments, including precipitation change, and also found no overall effect of precipitation on species richness. However, standard meta-analyses on reported effect sizes cannot disentangle complex biodiversity responses, such as the effects of the treatments on underlying components of abundance, evenness and aggregation that ultimately results in scale-dependent emergent patterns of species richness^11–13^. Ideally, a synthesis would explicitly consider (i) the magnitude and direction of the precipitation manipulation^8^; (ii) multiple aspects of biodiversity across multiple spatial scales^12^ and, (iii) information on background climate conditions, such as ambient levels of precipitation, which might influence how plant communities respond to precipitation change^10,14,15^.

Experiments vary widely in the magnitude and direction of precipitation manipulations^8^, and we expect that biodiversity responses will increase with the magnitude of the manipulation and depend on whether precipitation is experimentally augmented or decreased^16^. Experiments that reduce precipitation and those that increase precipitation differ methodologically (could have different artifacts, efficacy) and biologically (inducing germination from the seed bank vs. inducing mortality)^17^, and could therefore cause a non-linear relationship between the magnitude of precipitation manipulation (which ranges from highly negative to highly positive) and biodiversity. Biodiversity responses to environmental factors often vary across spatial grains of investigation^11^, due to changes in the abundance and composition of individuals within and across communities^12^, and it is reasonable to expect that precipitation will influence the abundance, dominance, and spatial turnover of species.

Plant productivity in dryer ecosystems tends to be more responsive to precipitation than less water-limited systems^14–16,18–21^. However, productivity is often a poor predictor of species richness^22^, and thus changes in productivity in response to climatic factors doesn’t necessarily coincide with changes in biodiversity^23^, due to scale-dependent biodiversity responses^11,12,24,25^. Nevertheless, plants located in arid ecosystems with low annual precipitation may show more dramatic biodiversity responses, since these communities are already facing hard physiological limitations to water availability^26^.

Here, we synthesize primary data from studies that experimentally manipulated precipitation in terrestrial ecosystems and measured the response of plant communities. For each study, we identify the intensity and direction of precipitation manipulation and the background environmental conditions at the study site, and then examine the scale-explicit response of species richness and relative abundances to these manipulations^11,12^. To accomplish this, we required studies that contained information on the sampling effort and relative abundances of plant species^12^. In all, we were able to acquire such detailed data from 72 experiments, embedded in 34 plant communities contained within 23 studies (see “Methods” section). These studies came primarily from North America and Europe, but also included other biogeographical regions (Supplementary Table 1). Studies spanned a broad range of background precipitation levels (ranging from 225 to 1574 mm per year), including primarily arid, semi-arid and mesic sites (Supplementary Fig. 1). We show that effect of precipitation manipulation on plant diversity (i) increases with the magnitude of precipitation manipulation—independent of the direction, (ii) is strongest at smaller spatial scales (i.e., plot compared to site scales), and (iii) depends on the background climatic conditions (is stronger in drier compared to wetter environments).

## Results and discussion

### Diversity responses to precipitation change depend on scale

**Fig. 1:**
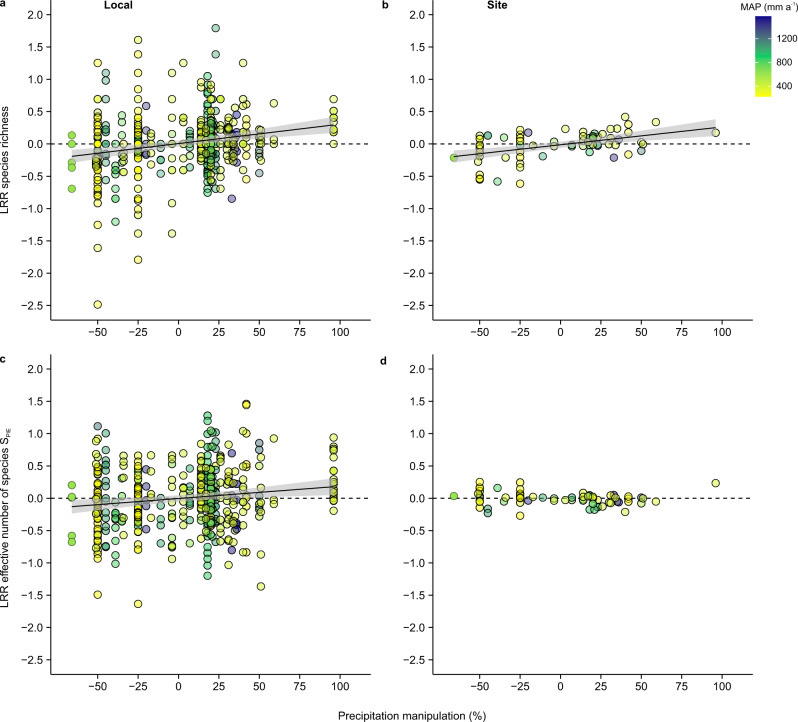
Responses of plant diversity to precipitation manipulation at local and site scales. Effect of the magnitude of precipitation manipulation on the log response ratio (LRR) of species richness (**a**, **b**) and effective number of species—S_PIE_ **c**, **d**) at the local scale (i.e., plot scale; **a**, **c**) and site scale (**b**, **d**). Data points represent log response ratios of original data (*n* = 462 at the local, *n* = 72 at the site scale) and colors indicate the background mean annual precipitation (MAP). The linear regressions (mean and 95% confidence intervals) are based on predicted values of the simplest linear mixed effect model including magnitude of precipitation manipulation (Supplementary Tables 2 and 3).

**Fig. 2:**
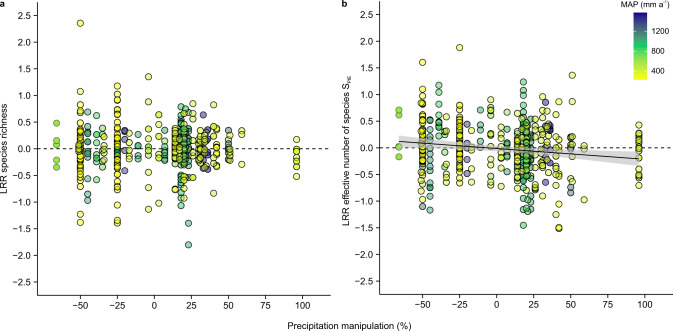
Responses of plant diversity to precipitation manipulation at the turnover scale. Effect of the magnitude of precipitation manipulation on the log response ratio (LRR) of species richness (**a**) and effective number of species—S_PIE_ (**b**) at the turnover scale (i.e., plot to plot scale). Data points represent log response ratios of original data (*n* = 462) and colors indicate the background mean annual precipitation (MAP). The linear regressions (mean and 95% confidence intervals) are based on predicted values of the simplest linear mixed effect model including magnitude of precipitation manipulation (Supplementary Tables 2 and 3).

At both the local scale (Fig. 1a) and site scale (Fig. 1b), species richness decreased with experimental decreases in precipitation, and increased with experimental increases in precipitation (conditional(c) *R*^2^ local = 0.14; c*R*^2^ site = 0.29, Supplementary Tables 2 and 3). One of the factors leading to the increase in species richness with precipitation was that treatments with higher levels of precipitation also had higher evenness across the more common species in the community (i.e., the effective number of species S_PIE_, Fig. 1c, c*R*^2^ = 0.13). However, this effect was tempered at larger spatial grains (Fig. 1d and Supplementary Tables 2 and 3). While consistent with previous meta-analyses^5,7,10^ and a recent observational study in California grasslands^15^, our synthesis allows us to gain deeper insights by pinpointing exactly how and where communities responded to experimental precipitation change. For example, larger changes in biodiversity at smaller spatial grains were primarily associated with increases in the total cover of plants (Supplementary Fig. 2a, c*R*^2^ = 0.21, Supplementary Tables 2 and 3). Likewise, the generally weaker effects of precipitation manipulations at larger spatial grains were primarily associated with a declining plot to plot variability in the shape of the species abundance distribution between treatments with low compared to higher precipitation (indicated by the negative effect sizes for the effective number of species S_PIE_, with increases in precipitation at the turnover scale; Fig. 2, c*R*^2^ = 0.14, Supplementary Tables 2 and 3). We found limited evidence of a non-linear relationship between precipitation manipulation and the effect size of biodiversity response variables (see “Methods” section, Supplementary Tables 4 and 5), indicating that even though there are different artifacts and biological responses possible between treatments that experimentally increase vs. decrease precipitation^17^, these effects were not large enough to change the slope of biodiversity responses.

### Diversity responses are strongest in dry communities

**Fig. 3:**
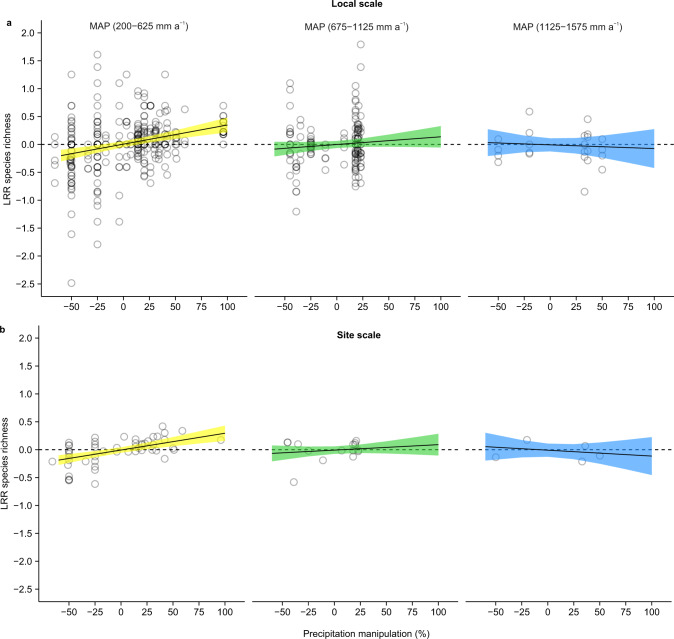
Climate-dependent effect of precipitation manipulation on plant species richness at local and site scales. Predictor effect plot of the sensitivity of the log response ratio of species richness at the local scale (**a**) and site scale (**b**) to manipulations in the magnitude of precipitation manipulation (%) depending on the range of background mean annual precipitation (MAP). Parameter estimates (mean and 95% confidence intervals) to create this figure are obtained from the simplest model including the interaction between magnitude of precipitation manipulation and MAP (Supplementary Tables 2 and 3). Different colors represent different ranges in background MAP: yellow, 200–675 mm a^−1^ (*n* = 300 at the local scale and *n* = 52 at the site scale); green, 675–1125 mm a^−1^ (*n* = 139 at the local scale and *n* = 15 at the site scale); blue, 1125–1575 mm a^−1^ (*n* = 23 at the local scale and *n* = 5 at the site scale). Data points represent the log response ratios of original data.

We found species richness had a steeper positive relationship with the precipitation manipulation in environments with drier mean annual precipitation relative to those in wetter environments at both small and large spatial grains (Supplementary Tables 2 and 3 and Fig. 3a, b, c*R*^2^ local = 0.16; c*R*^2^ site = 0.30). Most likely these climate-dependent effects of precipitation manipulation on species richness were primarily associated with stronger changes in the total cover of plants (Supplementary Fig. 3, c*R*^2^ = 0.24), at least on the local scale. When we used potential evapotranspiration (PET) instead of mean annual precipitation, results were largely similar, but PET significantly changes the slope of the evenness across the more common species at the local scale (i.e., effective number of species—S_PIE_; Supplementary Fig. 4, c*R*^2^ = 0.14, Supplementary Table 6). Specifically, plant communities in ecosystems with high potential evapotranspiration were more strongly affected by precipitation treatments than those with lower potential evapotranspiration (Supplementary Fig. 4).

### Diversity responses do not depend on life history

Mechanisms underlying these differential responses from drier to wetter environments may involve stronger responses of dryer communities with different dominant life history strategies (monocarpic vs. polycarpic), and associated differences in seed bank dynamics (i.e., the higher germination from a dormant seed bank and drought-induced seed dormancy in systems dominated by monocarps)^26^. We indeed found that aridity (if defined by PET) was related with higher probability of the dominant species of being monocarpic, i.e., annual or biennial (Chisq = 11.7, *P* < 0.001, Supplementary Fig. 5). However, communities dominated by monocarpic species showed equally strong responses to precipitation manipulation compared to communities dominated by polycarpic species across scales (monocarpic vs. polycarpic, all *P* > 0.10). This suggests the possibility that the stronger biodiversity responses of dryer communities might be rather driven by longer-term species losses due to mortality, and species gains due to immigration from the species pool.

Furthermore, shifts in the relative importance of abiotic versus biotic factors that control co-existence in these environments could also play a role in explaining the differences in the responses of drier and wetter communities^27^. For example, at drier sites with generally sparse vegetation, added precipitation can increase biodiversity by increasing the possibility of establishment on open patches^14^. At wetter sites with denser vegetation, biotic interactions (e.g., aboveground competition, herbivory), may be relatively more important and thus dilute responses of biodiversity to precipitation^28,29^. However, we have much higher certainty for how biodiversity changes in dry and warmer ecosystems compared to wetter and colder ecosystems, such as tropical/subtropical systems and tundra, due to low sample sizes in the latter systems (Supplementary Fig. 1). This is unfortunate, as both tropical and tundra ecosystems are highly threatened by other global change drivers (i.e., temperature increase and land use change), which may act in combination with precipitation change^7^, and we encourage future studies to fill this knowledge gap.

Unlike other recent meta-analyses^7,10^ our analyses accounting for a number of variables that differ across studies is able to detect significant and context-dependent effects of precipitation change on biodiversity. However, we note that we can still only explain a small amount of variation in biodiversity responses. This suggests that other variables that we do not include are also important, such as differences across experiments in soil conditions or biotic interactions. To achieve a more complete understanding of the effects of precipitation change on plant biodiversity, we suggest distributed experiments that standardize many of the factors that we included as fixed or random effects. However, we note that even globally distributed ecological experiments (e.g., NutNet) report *R*^2^ values within the same range as ours (0.10 and 0.25, see Harpole et al.^30^). We also suggest that there are many important additional factors that, if measured consistently and reported across studies, could be incorporated into future synthetic analyses.

Our results show environment-dependent effects of precipitation change on biodiversity, which may have important implications for defining management strategies for mitigating and adapting to global climate change. Furthermore, we show that these differential responses became evident only when spatial grain and magnitude of experimental manipulation were explicitly taken into account. While previous studies have suggested that temperature-limited ecosystems are particularly vulnerable to temperature increases with climate change, our synthesis shows that dryland ecosystems are particularly vulnerable to changes in precipitation patterns associated with climate change. This likely emerged because experimentally increased levels of precipitation typically increased the possibility of establishment of species from the species pool into open patches, whereas experimental reductions in precipitation in these already dry communities most likely led to higher mortality and thus a decline in species richness. This more pronounced effect of precipitation change on biodiversity in drylands is likely to represent a threat to these ecosystems^31^, which cover ~40% of the earth’s surface^32^, accommodate unique biological diversity^33^, and provide essential functions and services for at least one third of the human population worldwide^20,34^.

## Methods

### Literature review and characteristics of the experiments

We conducted a systematic literature research in the Web of Science and Google Scholar by using the search terms (precip* change OR precip* OR water OR irrigation OR drought OR climate change) AND (biodiversity OR rich* OR diversity OR abundance OR evenness) AND (plant community OR plant*AND terr* ecosystems) AND (field experiment OR climate change experiment OR climate manipulation OR simul* climate change). This literature search yielded around 1700 published studies from which we screened the title and abstracts. We also followed relevant references from the papers and from systematic reviews and meta-analysis, which yielded 20 additional studies (Supplementary Fig. 6). Studies were included if they manipulated precipitation in field experiments of terrestrial ecosystems and measured abundances of the plant species of the considered plant community (cover (%), biomass (g), or point intercept). We excluded experiments that were not in natural or semi-natural conditions (e.g., greenhouse, pot, or mesocosm experiments), as well as small-scale experiments that manipulated the numbers of species in each plot via seeding and weeding, because they excluded the possibility of immigration from the regional species pool. With these criteria, we identified 139 studies that were potentially appropriate, and then we determined whether they provided explicit data on species abundances for each plot/replication (e.g., in data repositories or supplementary material). We excluded datasets for which abundances were provided only at the level of functional groups. When data were not available, we inquired whether authors could provide the necessary data. In the end, our final dataset included 72 precipitation manipulation experiments from 23 studies (Supplementary Table 1). The choices of what we consider an experiment in this analysis was based on the choices made by the authors of the studies. For example, in several studies, similar experimental manipulations were performed in different sites and/or communities (e.g., lowland vs. mountain grassland; grassland at nutrient-poor vs. nutrient rich soil) and by using different types of precipitation manipulations (e.g., different frequency of events). All these experimental manipulations were considered as separate experiments, but nested hierarchically within study in all subsequent analyses.

We extracted information about the location of the experiment (i.e., latitude and longitude), the duration of the experiment (number of years) and the magnitude of experimental manipulation relative to the mean annual precipitation (%). We extracted climate variables from the Chelsa database^35^ (version 1.2) in order to have a consistent measure among studies. When community responses to climate manipulations were followed across multiple years and different seasons, we only used the endpoint of the time series at peak biomass for our analysis.

### Biodiversity response variables

We investigated the effects of precipitation manipulation on multiple response variables and scales^12,36^. Specifically, for each experiment, we measured the following:

- As a measure of how densely the plot was vegetated, we calculated the sum of the total abundances per sampling unit either from the absolute abundances (%) or from the total number of hits a plant touches the pin per plot (we could not estimate this measure from four of the 23 studies in our synthesis, as these data were not available).
- We calculated the total number of species—species richness (*S*)—at two scales. We calculated local (plot scale) *S* for each replicate in each treatment as the average number of species of species in a single plot/replicate (range of plot size: 0.08–2.5 m^2^). We calculated rarefied regional (treatment scale) *S* as the combination of all species observed in a given treatment/site combination (range of regional area: 0.4–38 m^2^) rarefied to the minimum number of replicates (*n* = 3)^37^. Because S does not include measures of relative abundance, it provides comparatively more weight to rarer relative to more common species.
- As a measure of diversity that explicitly weights common species more than rare species, we used Hurlbert’s probability of interspecific encounter (PIE)^38^ as a relatively unbiased measure of change in the dominance patterns of common species. To facilitate comparisons with species richness, we converted PIE to an effective number of species ${\rm{ENS}}_{\rm{PIE}}=1/{\sum }_{i=1}^{S}{{{\rm{pi}}}}^{2}$, where *S* is the total number of species and pi the proportion of each species *i* and we refer to this index as *S*_PIE_^12,39^. In the unlikely case that all species in a community are of equal relative abundance, *S* and *S*_PIE_ are the same, whereas as the community becomes more uneven, *S*_PIE_ becomes lower than *S*. As with *S*, we calculated *S*_PIE_ as the average from each replicate within a treatment and as a total from all replicates in that treatment.
- To elucidate treatment-level effects on patterns of community compositional differences among replicates within treatments (i.e., the turnover scale), we calculated the ratio of both *S* and *S*_PIE_ at the local (average of replicates) and site scales (total of replicates for *S*_PIE_ and bootstrapped mean based on 1000 iterations for rarefied richness *S*). This resulting ratio (equivalent to Whittaker’s diversity for *S*), captures differences in the spatial heterogeneity in species composition among treatments for all species (when calculated for *S*) and for primarily the most common species (when calculated for *S*_PIE_).

Rarefied richness was calculated using the iNext package^40^, *S*_PIE_ was calculated using the inverse of the Simpson index in the vegan package^41^. To calculate an effect size of the precipitation treatment, we used the log response ratio (LRR; LRR = ln (treatment) – ln (control)) as effect size^11,42^. Separate LRRs were calculated for each of the biodiversity components described above. A negative effect size indicates a decrease in the response variable due to the precipitation treatment, while a positive effect size indicates an increase in that response variable due to the treatment.

### Statistical analysis

To account for the non-independence of experiments at the different sites within a study, as well as differences in the experimental designs, we used linear mixed effect models with a hierarchical error structure^43^. Model selection was performed based on the Akaike information criterion for small sample sizes (AICc^44^). Mean effect sizes and 95% confidence intervals were bootstrapped using 1000 iterations and extracted from the simplest model including that factor^45^ (Supplementary Table 3). We consider an effect size as significant if the 95% CI is not overlapping the zero line. All analyses were performed in R version 4.0.3^46^. Visualizations of results were performed using the packages “ggplot2” and “effects”^47,48^.

Main analysis (Supplementary Table 2): For each response variable/scale combination, we used the interaction of block (for local and turnover scale only), site and study as random effect. The random term block was included in the analysis as denoted by the authors in the original study (e.g., in a randomized block design). If studies had no block as part of their design plots were treated as if all of them belonged to the same block. We used linear mixed models to test how the duration (years) of the experiments and the direction and magnitude of the precipitation manipulation (delta *P*, %) affected the LRR of species richness and effective number of species (*S*_PIE_), and how this in turn depended on mean annual precipitation (MAP, mm a^−1^) of the experimental location. All co-variates were scaled to the mean and two standard deviations before running the models^49^.

PET and experimental direction (Supplementary Tables 4–6): We tested whether the patterns were similar if a water-energy variable, specifically potential evapotranspiration (PET, kg a^−1^), was used instead of MAP (Supplementary Table 6). To test for potential asymmetric responses in the effect sizes due to precipitation increase or decrease, we performed two tests: (1) we tested for a non-linear term of delta *P* on the response variables (Supplementary Table 4), and (2) we included the interaction between delta *P* and the direction of manipulation into the model to test for different slopes in these two (non-overlapping) groups of data (Supplementary Table 5). The linear term performed similarly well compared to the quadratic term in all models (Supplementary Table 2 vs. Table 4), except for the effective number of species (*S*_PIE_) at the gamma scale, where the quadratic term performed better (Supplementary Fig. 7). Similarly, the interaction between the magnitude of manipulation and direction of manipulation was usually not among the best predictors (Supplementary Table 5).

Life history: To test whether plants with different life histories occur in sites with different MAP/PET and if these life history differences explain community responses, we extracted the most dominant species at each field site, categorized whether it was monocarpic (annual or biennial = 1), or polycarpic (perennial = 0), and tested if the probability of being monocarpic was related to MAP/PET using a mixed effects logistic regression with study as random term. Next, we tested whether systems dominated by monocarpic species differed in the effect sizes of precipitation manipulation on biodiversity response variables using mixed effects models with study as random term, life history as a fixed factor with two groups (monocarpic vs. polycarpic) and effect size of biodiversity response variables (log response ratio) at local and regional scales.

Effects of plot size and sample size: We examined if including plot size (m^2^) as a separate random effect or weighting the analysis by the square root of the sample size qualitatively influences the results (they did not; results not shown). Following an approach by Spake et al.^13^, we checked for study-level scale dependence that could obscure our synthesis by inspecting ubiquitous co-dependencies between the mean/variance in the effect size of our biodiversity response variables with sample size and plot size (Supplementary Fig. 8), as well as the covariation between the moderators with variance, sample size, and plot size (Supplementary Fig. 9). We found a correlation between the variance in the effect size in species richness and plot size, which was mainly driven by the study with the smallest plot size (Supplementary Fig. 8). Furthermore, we found a correlation between plot size and PET, delta *P*, and duration (Supplementary Fig. 9), which were mainly driven by the studies with the biggest plot size at local or site level. Removal of these study did, however, not change the results qualitatively and thus they were kept in the analysis.

### Reporting summary

Further information on research design is available in the Nature Research Reporting Summary linked to this article.

## Supplementary information

Supplementary Information

Reporting Summary

## Data Availability

The dataset that support the findings of this study are publicly available on Figshare (10.6084/m9.figshare.14061260) and the climate data are publicly available on the Chelsa database (10.5061/dryad.kd1d4).

## Code availability

The associated analysis code is archived on Github (https://github.com/lotte-korell/precipitation-change-experiments-.git).
