# Supplementary material for: Responses of plant diversity to precipitation change are strongest at local spatial scales and in drylands
Source: Nat Commun. 2021 May 3;12:2489. doi: 10.1038/s41467-021-22766-0 (PMC8093425; doi:10.1038/s41467-021-22766-0)
Supplement: Supplementary file 1 — Supplementary Information [file 41467_2021_22766_MOESM1_ESM.pdf]

Supplementary information

Responses of plant diversity to precipitation change are strongest at local spatial scales and in drylands

Lotte Korell* et al.

*corresponding author: lotte.korell@ufz.de

Contents:

Supplementary Tables 1 to 6

Supplementary Figures 1 to 9

Supplementary References

Supplementary Tables

Supplementary Table 1: Details about experiments included in the analyses. Study indicates the ID of study, experiment indicates the ID of experimental manipulation included in the analysis whereas type of community/ manipulation describes the community type or manipulation type of the experimental manipulation where each unique combination of site, community/ manipulation type, and Δ P is considered one experiment. Lat and long indicate the latitude and longitude of the site, respectively. Mean annual precipitation (MAP, mm a-1), mean annual temperature (MAT, ° C) and potential evapotranspiration (PET, kg a^{-1}) indicated the climatic conditions of the experimental sites. Magnitude of precipitation manipulation relative to mean annual precipitation (Δ P), as well as the abundance measurement taken (cov= cover, bm = biomass, pi = point intercept), local-scale plot size (in square meters) and number of experimental units (replicates) and the duration of the experimental treatment (in years) are given. Reference include the citation to the study under the section `Supplementary References´ at the end of this document (+denotes studies where unpublished data was provided).

#study	#exp	type of cummunity/ manipulation	country	continent	lat	long	MAP	PET	Δ P	abund	size	N	duration	Reference
1	1	shrubland	Israel	Asia	31.71	35.06	611	1299	-66	bm	0.04	5	1.0	1
2	2	shrubland	North America	America	34.33	-106.97	253	1333	-50	cov	1.00	3	3.0	2
2	3	shrubland	North America	America	34.33	-106.97	253	1333	42	cov	1.00	3	3.0	2
2	4	grassland	North America	America	34.33	-106.97	253	1333	-50	cov	1.00	3	3.0	2
2	5	grassland	North America	America	34.33	-106.97	253	1333	42	cov	1.00	3	3.0	2
2	6	shrub-grassland	North America	America	34.33	-106.97	253	1333	-50	cov	1.00	3	3.0	2
2	7	shrub-grassland	North America	America	34.33	-106.97	253	1333	42	cov	1.00	3	3.0	2
3	8	mixed grass-prairie	North America	America	41.00	-104.00	361	1067	23	bm	1.00	3	2.0	3
3	9	mixed grass-prairie	North America	America	41.00	-104.00	361	1067	35	bm	1.00	3	2.0	3
3	10	mixed grass-prairie	North America	America	41.00	-104.00	361	1067	59	bm	1.00	3	2.0	3
4	11	grassland	North America	America	34.33	-106.72	291	1315	20	cov	1.00	5	10.0	4
5	12	harsh serpentine grassland	North America	America	38.87	-122.43	872	1157	18	cov	1.00	3	4.0	5
5	13	lush serpentine grassland	North America	America	38.87	-122.43	872	1157	18	cov	1.00	3	4.0	5
5	14	nonserpentine grassland	North America	America	38.87	-122.43	872	1157	18	cov	1.00	3	4.0	5
6	15	grassland	Great Britain	Europe	51.41	-0.65	640	670	7	cov	1.00	4	2.0	6
6	16	grassland	Great Britain	Europe	51.41	-0.65	640	670	-4	cov	1.00	4	2.0	6
7	17	semi-natural grassland	Switzerland	Europe	47.21	8.14	1522	759	33	bm	0.20	5	2.0	7
7	18	semi-natural grassland	Switzerland	Europe	47.12	8.14	1574	771	36	bm	0.20	6	2.0	7
8	19	limestone grassland	Great Britain	Europe	53.23	-1.90	1001	591	-11	pi	2.50	5	16.0	8
8	20	limestone grassland	Great Britain	Europe	53.23	-1.90	1001	591	7	pi	2.50	5	16.0	8
9	21	grassland/ low grazed	North America	America	39.25	-121.28	1034	1347	-45	cov	0.25	8	2.0	9
9	22	grassland/ low grazed	North America	America	39.25	-121.28	1034	1347	-45	cov	0.25	8	2.0	9
9	23	grassland/high grazed	North America	America	39.25	-121.28	1034	1347	23	cov	0.25	8	2.0	9
9	24	grassland/high grazed	North America	America	39.25	-121.28	1034	1347	23	cov	0.25	8	2.0	9
10	25	grassland	North America	America	33.62	-117.76	285	1284	31	bm	0.11	5	0.2	10
11	26	lowland grassland	Czech Republic	Europe	48.82	16.00	495	783	-50	cov	0.50	4	2.0	11
11	27	lowland grassland	Czech Republic	Europe	48.82	16.00	495	783	50	cov	0.50	4	2.0	11
11	28	mountain grassland	Czech Republic	Europe	49.50	18.53	1198	665	-50	cov	0.50	4	2.0	11

#study	#exp	type of cummunity/ manipulation	country	continent	lat	long	MAP	PET	Δ P	abund	size	N	duration	Reference
11	29	mountain grassland	Czech Republic	Europe	49.50	18.53	1198	665	50	cov	0.50	4	2.0	11
12	30	semi-natural grassland	Germany	Europe	50.44	11.48	913	670	-25	cov	2.00	19	1.0	12
13	31	shortgrass steppe	North America	America	40.83	-104.75	372	1081	96	bm	0.25	10	14.0	13
14	32	floodplain meadow	Germany	Europe	52.78	12.18	537	697	14	cov	0.25	7	3.0	14, +
14	33	floodplain meadow	Germany	Europe	53.32	10.95	629	665	14	cov	0.25	7	3.0	14, +
15	34	semi-natural grassland	Switzerland	Europe	46.85	6.58	1427	700	-20	cov	0.25	5	3.0	15
16	35	coastal sand dune/ weekly	Spain	Europe	36.82	-2.25	225	949	-25	bm	0.08	5	0.5	16
16	36	coastal sand dune/ by-weekly	Spain	Europe	36.82	-2.25	225	949	-25	bm	0.08	5	0.5	16
16	37	coastal sand dune/ monthly	Spain	Europe	36.82	-2.25	225	949	-25	bm	0.08	5	0.5	16
16	38	coastal sand dune/ weekly	Spain	Europe	36.82	-2.25	225	949	-50	bm	0.08	5	0.5	16
16	39	coastal sand dune/ by-weekly	Spain	Europe	36.82	-2.25	225	949	-50	bm	0.08	5	0.5	16
16	40	coastal sand dune/ monthly	Spain	Europe	36.82	-2.25	225	949	-50	bm	0.08	5	0.5	16
16	41	abandoned field/ weekly	Spain	Europe	37.00	-2.43	311	1047	-25	bm	0.08	5	0.5	16
16	42	abandoned field/ biweekly	Spain	Europe	37.00	-2.43	311	1047	-25	bm	0.08	5	0.5	16
16	43	abandoned field/ monthly	Spain	Europe	37.00	-2.43	311	1047	-25	bm	0.08	5	0.5	16
16	44	abandoned field/ weekly	Spain	Europe	37.00	-2.43	311	1047	-50	bm	0.08	5	0.5	16
16	45	abandoned field/ biweekly	Spain	Europe	37.00	-2.43	311	1047	-50	bm	0.08	5	0.5	16
16	46	abandoned field/ monthly	Spain	Europe	37.08	-2.35	234	1041	-25	bm	0.08	5	0.5	16
16	47	annual salt grassland/weekly	Spain	Europe	37.08	-2.35	234	1041	-25	bm	0.08	5	0.5	16
16	48	annual salt grassland/biweekly	Spain	Europe	37.08	-2.35	234	1041	-25	bm	0.08	5	0.5	16
16	49	annual salt grassland/monthly	Spain	Europe	37.08	-2.35	234	1041	-50	bm	0.08	5	0.5	16
16	50	annual salt grassland/weekly	Spain	Europe	37.08	-2.35	234	1041	-50	bm	0.08	5	0.5	16
16	51	annual salt grassland/monthly	Spain	Europe	37.08	-2.35	234	1041	-50	bm	0.08	5	0.5	16
17	52	grassland	North America	America	42.83	-104.90	419	992	30	bm	1.50	5	7.0	17, +
18	53	grassland	North America	America	40.12	-105.30	409	1059	26	cov	0.25	10	3.0	18
18	54	grassland	North America	America	40.12	-105.30	409	1059	-4	cov	0.25	10	3.0	18
18	55	grassland	North America	America	40.12	-105.30	409	1059	40	cov	0.25	10	3.0	18
18	56	grassland	North America	America	40.12	-105.30	409	1059	3	cov	0.25	10	3.0	18
19	57	old-field meadow	Canada	America	44.63	-76.33	827	786	-39	cov	0.50	10	4.0	19
19	58	old-field meadow	Canada	America	44.63	-76.33	827	786	21	cov	0.50	10	4.0	19
20	59	tallgrass prairie	North America	America	39.11	-96.62	724	1100	-35	cov	1.00	5	3.0	20
21	60	shortgrass/small events	North America	America	41.40	-105.27	312	925	20	bm	1.00	10	2.0	21
21	61	shortgrass/ large events	North America	America	41.40	-105.27	312	925	20	bm	1.00	10	2.0	21
21	62	mixed grassland/small events	North America	America	46.52	-106.63	332	982	14	bm	1.00	10	2.0	21
21	63	mixed grassland/large events	North America	America	46.52	-106.63	332	982	14	bm	1.00	10	2.0	21
21	64	tallgrass/ small events	North America	America	39.15	-96.92	765	1103	20	bm	1.00	10	2.0	21
21	65	tallgrass/ large events	North America	America	39.15	-96.92	765	1103	20	bm	1.00	10	2.0	21
22	66	steppe	China	Asia	42.03	116.28	372	798	31	cov	1.00	6	4.0	22
23	67	steppe	China	Asia	42.03	116.28	372	798	-17	cov	1.00	6	3.0	23
23	68	steppe	China	Asia	42.03	116.28	372	798	-34	cov	1.00	6	3.0	23
23	69	steppe	China	Asia	42.03	116.28	372	798	-51	cov	1.00	6	3.0	23
23	70	steppe	China	Asia	42.03	116.28	372	798	17	cov	1.00	6	3.0	23
23	71	steppe	China	Asia	42.03	116.28	372	798	34	cov	1.00	6	3.0	23
23	72	steppe	China	Asia	42.03	116.28	372	798	51	cov	1.00	6	3.0	23

Supplementary Table 2: Summary statistics for the highest-ranked mixed effects model (delta AICc < 2) for the response variables species richness, effective number of species (S_{PIE}) and total vegetation cover across scales (local scale, turnover scale, site scale). Degrees of freedom (df), log likelihood (logLik), delta AICc, AICc weights and conditional variance explained (cR^2) and marginal variance explained (mR^2) are shown. Models include following variables: linear term of the magnitude of precipitation manipulation (delta P, %), study duration (years), and mean annual precipitation (MAP, mm a-1) '+' indicates an additive effect and ':' interactive effects between variables.

response	scale	moderators	df	logLik	AICc	delta	weight	cR^2	mR^2
Species richness	local	delta P + MAP + delta P : MAP	6	-267.12	546.42	0.00	0.41	0.162	0.083
		delta P + MAP + duration + delta P : MAP	7	-266.09	546.42	0.01	0.40	0.158	0.082
		delta P	4	-269.92	547.94	1.52	0.19	0.144	0.060
	turnover	-	3	-235.34	476.74	0.00	0.44	0.071	0.000
		delta P	4	-235.15	478.39	1.65	0.19	0.074	0.001
		duration	4	-235.23	478.55	1.81	0.18	0.074	0.001
	site	delta P + MAP + delta P : MAP	6	21.24	-29.18	0.00	0.51	0.302	0.348
		delta P + MAP + duration + delta P : MAP	7	21.76	-27.77	1.41	0.25	0.312	0.353
		delta P	4	18.10	-27.60	1.58	0.23	0.292	0.225
S_{PIE}	local	delta P	4	-283.30	574.69	0.00	0.56	0.130	0.023
		delta P + MAP	5	-283.18	576.50	1.81	0.23	0.134	0.023
		delta P + duration	5	-283.25	576.63	1.94	0.21	0.132	0.022
	turnover	delta P + duration	5	-295.73	601.60	0.00	0.30	0.148	0.026
		delta P	4	-296.83	601.75	0.15	0.28	0.145	0.025
		delta P + MAP + duration + delta P : MAP	7	-294.22	602.69	1.09	0.17	0.144	0.039
		delta P + MAP + duration	6	-295.48	603.15	1.55	0.14	0.144	0.028
		delta P + MAP	5	-296.68	603.49	1.90	0.12	0.147	0.027
	site	duration	4	63.30	-117.80	0.00	0.25	0.322	0.000
		delta P + duration	5	64.20	-117.17	0.62	0.18	0.368	0.044
		-	3	61.73	-116.98	0.82	0.17	0.343	0.019
		duration + MAP	5	64.10	-116.97	0.83	0.17	0.337	0.057
		delta P + duration + MAP	6	65.06	-116.37	1.43	0.12	0.362	0.083
		MAP	4	62.44	-116.08	1.71	0.11	0.322	0.039
Total abundance	local	delta P + MAP + delta P : MAP	6	-447.32	906.84	0.00	0.68	0.247	0.183
		delta P	4	-450.12	908.33	1.49	0.32	0.215	0.155
		delta P	4	-27.00	62.64	0.00	1.00	0.291	0.282

Supplementary Table 3: Mean bootstrapped effect sizes (log response ratios = LRR) and 95% confidence intervals (upper and lower CI) for the response variables species richness, effective number of species (S_{PIE}) and total abundance from the simplest model including the fixed effect of magnitude of precipitation manipulation (delta P), mean annual precipitation (MAP), potential evapotranspiration (PET), study duration, (duration), interaction magnitude of precipitation manipulation and mean annual precipitation (delta P : MAP), interaction magnitude of precipitation manipulation and potential evapotranspiration (delta P : PET), and the interaction of the magnitude of precipitation manipulation and direction of manipulation (decreases: direction (decr. P): delta P; increases: direction (incr. P): delta P) across scales (local scale, turnover, site scale). Bold letters indicate effect sizes and CI that do not overlap zero and are thus considered significant. Grey shaded cells indicate values where the simplest model was included among the highest-ranked mixed effects models (delta AICc < 2) (Supplementary Table 2, Table 3, Table 6).

**moderators**		**Local scale**			**Turnover scale**		**Site scale**		
	model estimates	Species richness	S_{PIE}	Total abundance	Species richness	S_{PIE}	Species richness	S_{PIE}	Total abundance
grand mean	effect size	0.02	0.01	0.04	-0.01	-0.02	-0.01	-0.01	-0.04
	upper CI	0.07	0.06	0.13	0.03	0.03	0.05	0.02	0.07
	lower CI	-0.03	-0.04	-0.04	-0.05	-0.08	-0.07	-0.04	-0.15
delta P	effect size	**0.21**	**0.13**	**0.55**	-0.03	**-0.14**	**0.19**	-0.03	**0.44**
	upper CI	**0.29**	**0.22**	**0.70**	0.05	**-0.05**	**0.28**	0.02	**0.61**
	lower CI	**0.13**	**0.04**	**0.40**	-0.10	**-0.23**	**0.10**	-0.07	**0.27**
MAP	effect size	-0.03	-0.04	0.03	-0.01	-0.03	0.00	-0.05	0.09
	upper CI	0.08	0.08	0.24	0.09	0.09	0.12	0.01	0.35
	lower CI	-0.15	-0.16	-0.19	-0.11	-0.15	-0.13	-0.10	-0.17
PET	effect size	0.09	0.05	0.13	-0.02	-0.11	-0.02	0.00	-0.03
	upper CI	0.22	0.18	0.32	0.08	0.02	0.04	0.13	0.19
	lower CI	-0.02	-0.06	-0.06	-0.11	-0.23	-0.08	-0.11	-0.25
duration	effect size	-0.03	0.01	0.08	0.00	0.08	0.06	0.05	0.24
	upper CI	0.11	0.16	0.34	0.12	0.23	0.23	0.13	0.52
	lower CI	-0.18	-0.14	-0.18	-0.13	-0.08	-0.12	-0.04	-0.06
delta P : MAP	effect size	**-0.25**	-0.14	**-0.40**	0.03	0.14	**-0.24**	0.04	-0.30
	upper CI	**-0.05**	0.08	**-0.07**	0.22	0.37	**-0.07**	0.12	0.10
	lower CI	**-0.47**	-0.37	**-0.72**	-0.17	-0.10	**-0.44**	-0.06	-0.69
delta P : PET	effect size	0.02	**0.27**	0.24	-0.07	-0.17	0.10	0.03	0.10
	upper CI	0.28	**0.54**	0.66	0.17	0.10	0.35	0.14	0.15
	lower CI	-0.24	**0.01**	-0.17	-0.31	-0.44	-0.14	-0.07	-0.21
direction (decr. P): delta P	effect size	**0.20**	0.10	**0.54**	-0.01	-0.12	**0.20**	-0.03	**0.49**
	upper CI	**0.33**	0.24	**0.78**	0.11	0.02	**0.32**	0.03	**0.69**
	lower CI	**0.06**	-0.04	**0.32**	-0.13	-0.27	**0.09**	-0.09	**0.27**
direction (incr. P) : delta P	effect size	**0.22**	**0.16**	**0.56**	-0.04	**-0.16**	**0.18**	-0.02	**0.37**
	upper CI	**0.11**	**0.30**	**0.75**	0.07	**-0.02**	**0.32**	0.05	**0.62**
	lower CI	**0.35**	**0.04**	**0.37**	-0.15	**-0.29**	**0.04**	-0.09	**0.11**

Supplementary Table 4: Summary statistics for the highest-ranked mixed effects model (delta AICc < 2) for the response variables species richness, effective number of species (S_{PIE}) and total vegetation across scales (local scale, turnover scale, site scale). Degrees of freedom (df), log likelihood (logLik), delta AICc, AICc weights and conditional variance explained (cR^2) and marginal variance explained (mR^2) are shown. Models include following variables: linear and quadratic term of magnitude of precipitation manipulation (delta P, %), study duration (years), and mean annual precipitation (MAP, mm a-1). '+' indicates an additive effect and ':' interactive effects between variables

response	scale	moderators	df	logLik	AICc	delta	weight	cR^2	mR^2
Species richness	local	delta P + MAP + delta P : MAP	6	-267.12	546.42	0.00	0.29	0.162	0.083
		delta P + MAP + duration + delta P : MAP	7	-266.09	546.42	0.01	0.29	0.158	0.082
		delta P + delta P^2 + MAP + delta P : MAP	7	-266.56	547.37	0.96	0.18	0.162	0.082
		delta P	4	-269.92	547.94	1.52	0.13	0.144	0.060
		delta P + delta P^2 + MAP + delta P : MAP	8	-265.94	548.19	1.77	0.12	0.161	0.081
	turnover	-	3	-235.34	476.74	0.00	0.44	0.071	0.000
		delta P	4	-235.15	478.39	1.65	0.19	0.074	0.001
		delta P^2	4	-235.21	478.50	1.76	0.18	0.072	0.001
		duration	4	-235.23	478.55	1.81	0.18	0.074	0.001
	site	delta P + MAP + delta P : MAP	6	21.24	-29.18	0.00	0.42	0.302	0.348
		delta P + MAP + duration + delta P : MAP	7	21.76	-27.77	1.41	0.21	0.312	0.353
		delta P	4	18.10	-27.60	1.58	0.19	0.292	0.225
		delta P + delta P^2 + MAP + delta P : MAP	7	21.63	-27.51	1.67	0.18	0.354	0.310
S_{PIE}	local	delta P	4	-283.30	574.69	0.00	0.44	0.130	0.023
		delta P + delta P^2	5	-282.97	576.08	1.39	0.22	0.132	0.028
		delta P + MAP	5	-283.18	576.50	1.81	0.18	0.134	0.023
		delta P + duration	5	-283.25	576.63	1.94	0.17	0.132	0.022
	turnover	delta P + duration	5	-295.73	601.60	0.00	0.24	0.148	0.026
		delta P	4	-296.83	601.75	0.15	0.22	0.145	0.025
		delta P + MAP + duration + delta P : MAP	7	-294.22	602.69	1.09	0.14	0.144	0.039
		delta P + MAP + duration	6	-295.48	603.15	1.55	0.11	0.144	0.028
		delta P + delta P^2	5	-296.54	603.21	1.62	0.11	0.145	0.024
		delta P + MAP	5	-296.68	603.49	1.90	0.09	0.147	0.027
		delta P + delta P^2 + duration	6	-295.70	603.59	1.99	0.09	0.145	0.025
	site	delta P^2	4	71.39	-134.18	0.00	0.39	0.342	0.069
		delta P^2 + MAP	5	72.16	-133.42	0.76	0.27	0.345	0.096
		delta P + delta P^2	5	71.79	-132.66	1.52	0.18	0.350	0.077
		delta P^2 + duration	5	71.64	-132.38	1.80	0.16	0.353	0.074
Total abundance	local	delta P + MAP + delta P : MAP	6	-447.32	906.84	0.00	0.68	0.247	0.183
		delta P	4	-450.12	908.33	1.49	0.32	0.215	0.155
		delta P	4	-27.00	62.64	0.00	1.00	0.291	0.282

Supplementary Table 5: Summary statistics for the highest-ranked mixed effects model (delta AICc < 2) for the response variables species richness, effective number of species (S_{PIE}) and total vegetation cover across scales (local scale, turnover scale, site scale). Degrees of freedom (df), log likelihood (logLik), delta AICc, AICc weights and conditional variance explained (cR^2) and marginal variance explained (mR^2) are shown. Models include following variables: linear term of the magnitude of precipitation manipulation (delta P), direction of the manipulation (direction), study duration (years), and mean annual precipitation (MAP, mm a-1). '+' indicates an additive effect and ':' interactive effects between variables.

response	scale	moderators	df	logLik	AICc	delta	weight	cR^2	mR^2
Species richness	local	delta P + MAP + delta P : MAP	6	-267.12	546.42	0.00	0.28	0.162	0.083
		delta P + MAP + duration + delta P : MAP	7	-266.09	546.42	0.01	0.28	0.158	0.082
		delta P + MAP + duration + delta P : direction	7	-266.54	547.33	0.91	0.18	0.150	0.057
		delta P	4	-269.92	547.94	1.52	0.13	0.144	0.060
		delta P + MAP + duration + delta P : MAP + delta P : direction	8	-265.82	547.96	1.55	0.13	0.161	0.082
	turnover	-	3	-235.34	476.74	0.00	0.44	0.071	0.000
		delta P	4	-235.15	478.39	1.65	0.19	0.074	0.001
		duration	4	-235.23	478.55	1.81	0.18	0.074	0.001
	site	delta P + MAP + delta P : MAP	6	21.24	-29.18	0.00	0.42	0.302	0.348
		delta P + MAP + duration + delta P : MAP	7	21.76	-27.77	1.41	0.21	0.312	0.353
		delta P	4	18.10	-27.60	1.58	0.19	0.292	0.225
		delta P + MAP + duration + delta P : MAP + delta P : direction	7	21.56	-27.37	1.81	0.17	0.309	0.353
S_{PIE}	local	delta P	4	-283.30	574.69	0.00	0.43	0.130	0.023
		delta P + delta P: direction	5	-282.91	575.95	1.27	0.23	0.132	0.026
		delta P + MAP	5	-283.18	576.50	1.81	0.17	0.134	0.023
		delta P + duration	5	-283.25	576.63	1.94	0.16	0.132	0.022
	turnover	delta P + duration	5	-295.73	601.60	0.00	0.27	0.148	0.026
		delta P	4	-296.83	601.75	0.15	0.25	0.145	0.025
		delta P + MAP + duration + delta P : MAP	7	-294.22	602.69	1.09	0.16	0.144	0.039
		delta P + MAP + duration	6	-295.48	603.15	1.55	0.12	0.144	0.028
		delta P + MAP	5	-296.68	603.49	1.90	0.10	0.147	0.027
		delta P + duration + delta P : direction	6	-295.70	603.58	1.98	0.10	0.144	0.0255
	site	-	3	68.68	-131.00	0.00	0.17	0.322	0.000
		MAP	4	69.79	-130.99	0.02	0.17	0.322	0.039
		delta P	4	69.39	-130.19	0.81	0.11	0.343	0.019
		duration + MAP	5	70.54	-130.17	0.83	0.11	0.337	0.057
		delta P + duration + MAP	6	71.65	-130.00	1.00	0.10	0.362	0.083
		delta P + MAP	5	70.40	-129.89	1.12	0.10	0.345	0.057
		delta P + duration	5	70.34	-129.78	1.23	0.09	0.368	0.044
		duration	4	69.18	-129.76	1.24	0.09	0.345	0.018
		delta P + delta P : direction	5	70.02	-129.13	1.88	0.07	0.341	0.036
Total abundance	local	delta P + MAP + delta P : MAP	6	-447.32	906.84	0.00	0.68	0.247	0.183
		delta P	4	-450.12	908.33	1.49	0.32	0.215	0.155
		delta P	4	-27.00	62.64	0.00	1.00	0.291	0.282

Supplementary Table 6: Summary statistics for the highest-ranked mixed effects model (delta AICc < 2) for the response variables species richness, effective number of species (S_{PIE}) and total vegetation cover across scales (local scale, turnover scale, site scale). Degrees of freedom (df), log likelihood (logLik), delta AICc, AICc weights and conditional variance explained (cR^2) and marginal variance explained (mR^2) are shown. Models include following variables: linear term of the magnitude of precipitation manipulation (delta P, %), study duration (years), and potential evapotranspiration (PET, kg a-1) '+' indicates an additive effect and ':' interactive effects between variables.

response	scale	moderators	df	logLik	AICc	delta	weight	cR^2	mR^2
Species richness	local	delta P	4	-269.92	547.94	0.00	0.36	0.144	0.060
		delta P + PET	5	-269.08	548.30	0.37	0.30	0.146	0.062
		delta P + duration	5	-269.48	549.10	1.16	0.20	0.145	0.058
		delta P + duration + PET	6	-268.79	549.77	1.83	0.14	0.146	0.060
	turnover	-	3	-235.34	476.74	0.00	0.45	0.071	0.000
		delta P	4	-235.15	478.39	1.65	0.20	0.087	0.002
		duration	4	-235.23	478.55	1.81	0.18	0.074	0.001
		PET	4	-235.32	478.73	1.99	0.17	0.074	0.000
	site	delta P	4	18.10	-27.60	0.00	0.72	0.216	0.115
		delta P + PET	5	18.33	-25.76	1.84	0.28	0.294	0.219
S_{PIE}	local	delta P + PET + delta P : PET	6	-280.21	572.60	0.00	0.73	0.142	0.039
		delta P + PET + duration + delta P : PET	7	-280.15	574.55	1.95	0.27	0.143	0.037
	turnover	delta P + PET	5	-295.25	600.62	0.00	0.21	0.148	0.029
		delta P + PET + delta P : PET	6	-294.27	600.72	0.09	0.20	0.150	0.034
		delta P + PET + duration + delta P : PET	7	-293.36	600.96	0.34	0.18	0.150	0.034
		delta P + PET + duration	6	-294.46	601.11	0.49	0.16	0.145	0.029
		delta P + duration	5	-295.73	601.60	0.97	0.13	0.143	0.025
		delta P	4	-296.83	601.75	1.12	0.12	0.145	0.025
	site	-	3	64.04	-121.72	0.00	0.34	0.322	0.000
		duration	4	64.63	-120.66	1.07	0.20	0.343	0.019
		delta P	4	64.49	-120.37	1.35	0.18	0.343	0.019
		delta P + duration	5	65.41	-119.92	1.80	0.14	0.368	0.044
		PET	4	64.25	-119.90	1.82	0.14	0.328	0.001
Total abundance	local	delta P	4	-450.12	908.33	0.00	0.55	0.215	0.155
		delta P + duration	5	-449.97	910.07	1.74	0.23	0.217	0.158
		delta P + PET	5	-449.97	910.08	1.75	0.23	0.215	0.155
	site	delta P	4	-27.00	62.64	0.00	0.73	0.291	0.282
		delta P + PET	5	-26.82	64.61	1.97	0.27	0.293	0.283

Supplementary Figures

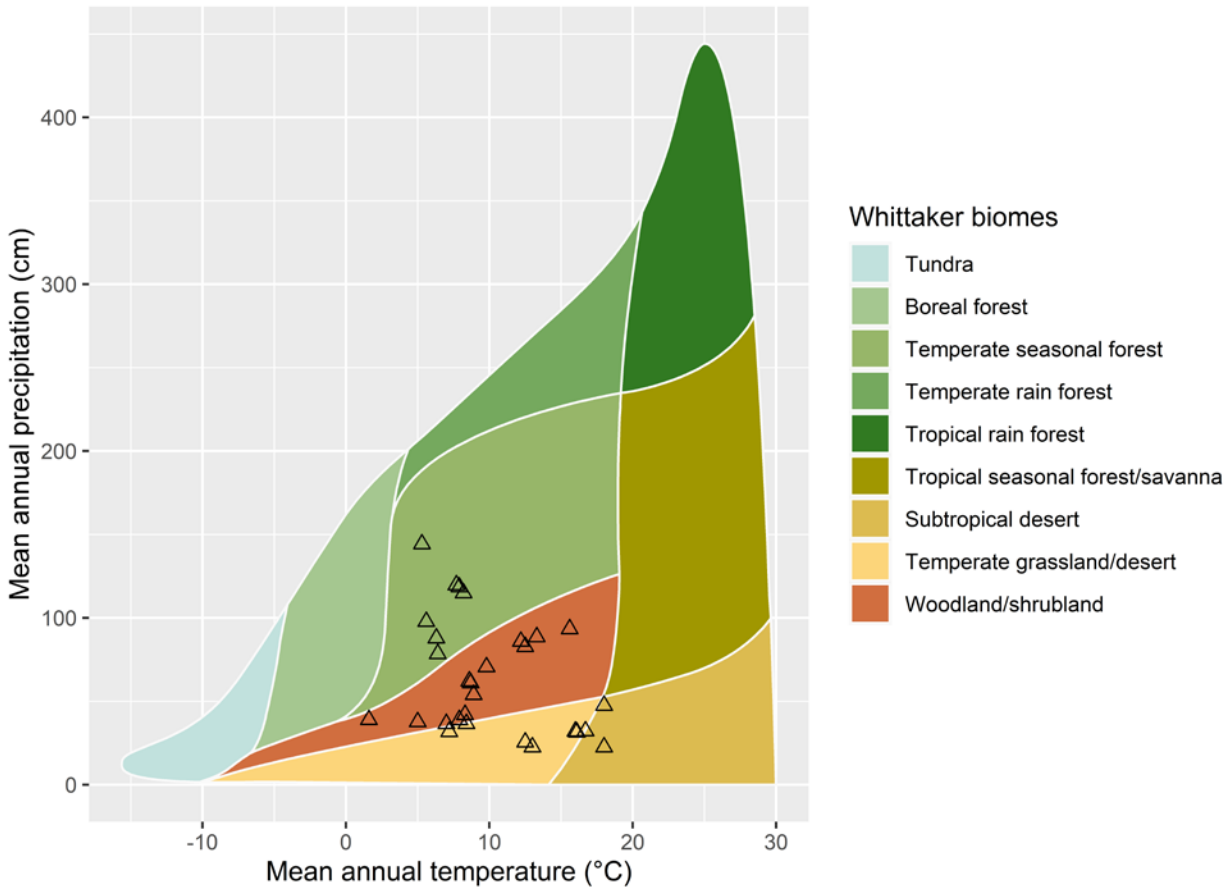

Supplementary Figure 1: Whittaker´s biome plot, defined by mean annual temperature (cm) and mean annual precipitation (°C).Triangles represent precipitation manipulation experiments.

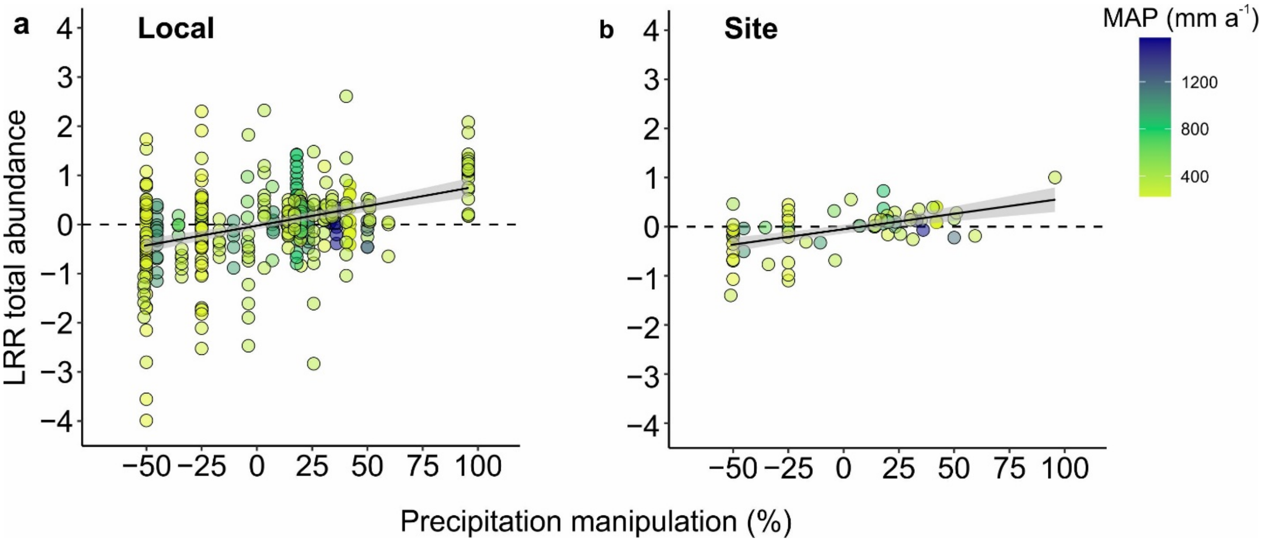

Supplementary Figure 2: Effect of the magnitude of precipitation manipulation on the log response ratio (LRR) of the total vegetation cover at the local scale (**a**) and site scale (**b**). Data points represent log response ratios of original data (n = 432 at the local scale and n = 69 at the site scale) and colors indicate the background climatic conditions; mean annual precipitation (MAP). The linear regressions (mean and 95% confidence intervals) are based on predicted values of the simplest linear mixed effect model including magnitude of precipitation manipulation (Supplementary Table 2).

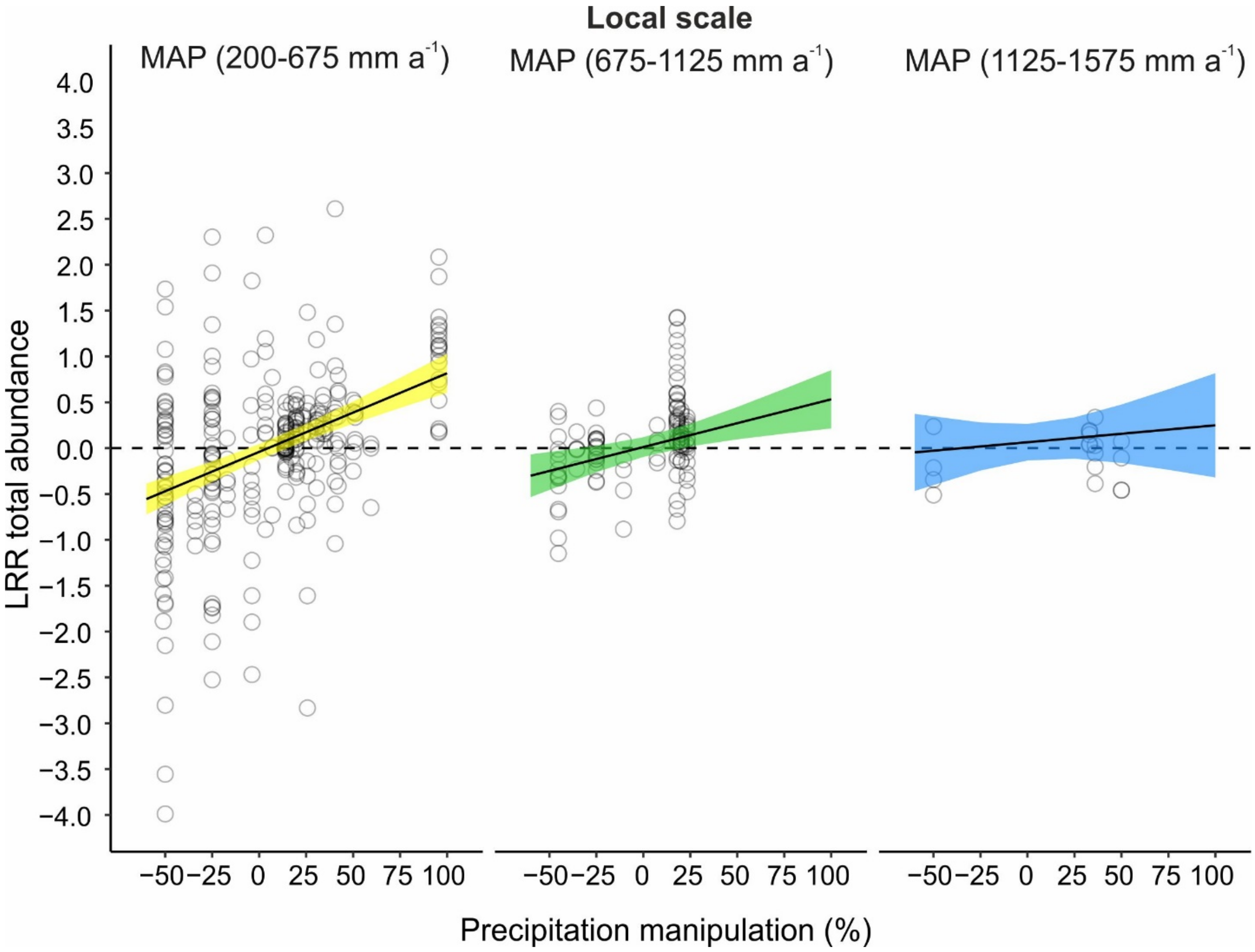

Supplementary Figure 3: Predictor effect plot of the sensitivity of the log response ratio of total abundance at the local scale to manipulations in the magnitude of precipitation manipulation (%) depending on the range of background mean annual precipitation (MAP). Parameter estimates (mean and 95% confidence intervals) to create this figure are obtained from the simplest model including the interaction between magnitude of precipitation manipulation and MAP (Supplementary Tables 2-3). Different colors represent different ranges in background MAP: yellow, 200-675 mm a-1 (n = 295); green, 675-1125 mm a-1 (n = 119); blue, 1125-1575 mm a-1 (n = 18). Data points represent the log response ratios of original data.

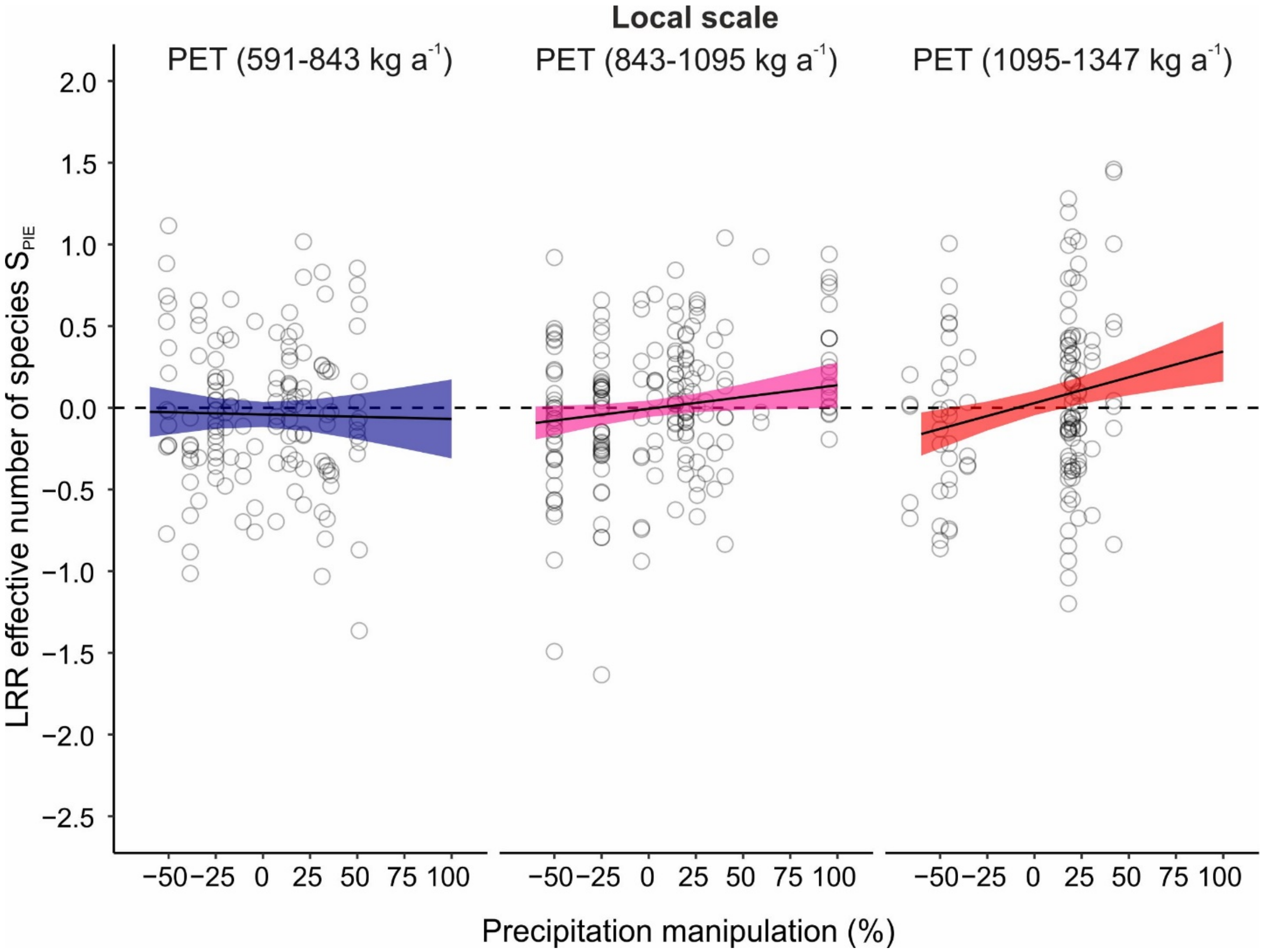

Supplementary Figure 4: Predictor effect plot of the sensitivity of the effect size (log response ratio) of the effective number of species S_{PIE} to manipulations in the magnitude of precipitation manipulation (%) depending on the range of background potential evapotranspiration (PET). Parameter estimates (mean and 95% confidence intervals) to create this figure are obtained from the simplest model including the interaction between magnitude of precipitation manipulation and PET (Supplementary Table 4). Different colors represent different ranges in background PET: blue, 591-843 kg a^{-1} (n = 144); pink, 843-1095 kg a^{-1} (n = 195); red, 1095-2347mm a-1 (n = 123). Data points represent the log response ratios of original data.

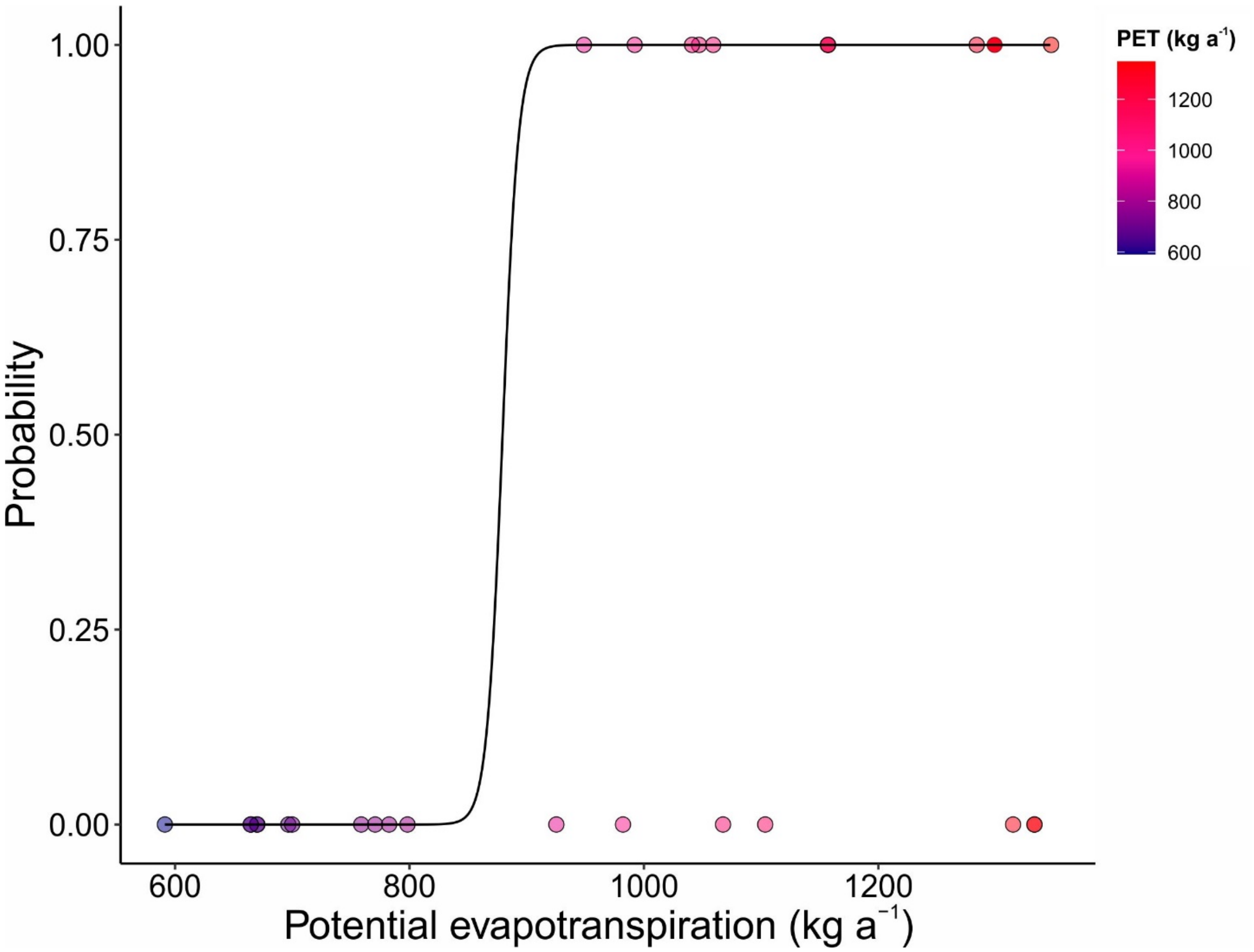

Supplementary Figure 5: Results of a mixed effects logistic regression showing the probability of the most dominant species within experimental site of being monocarpic in relation to the site-level potential evapotranspiration (PET). Data points represent site-specific values on the most dominant species to monocarpic (annual or biennial = 1), or polycarpic (perennial = 0).

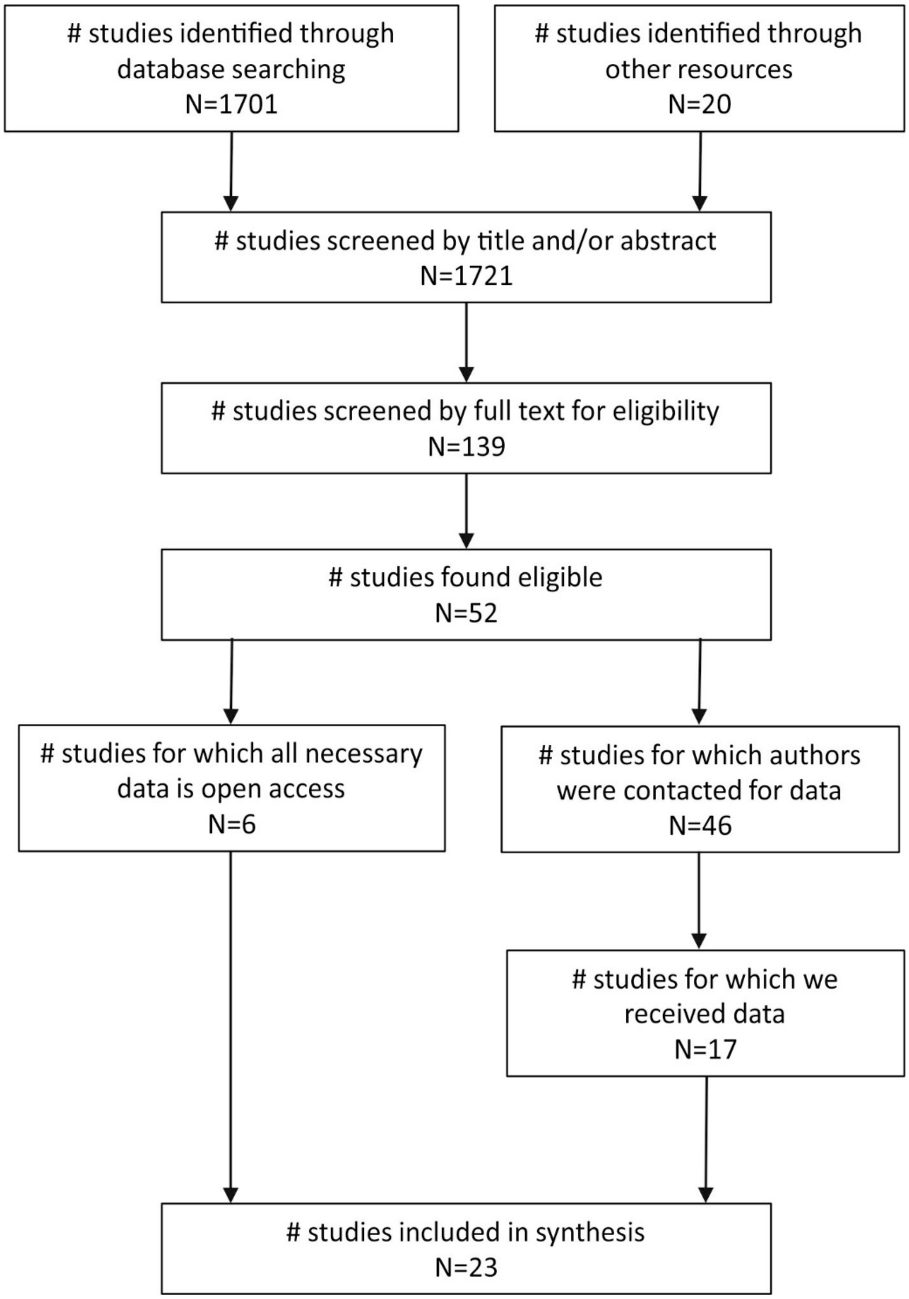

Supplementary Figure 6: Prisma diagram showing the different phases of the systematic literature review.

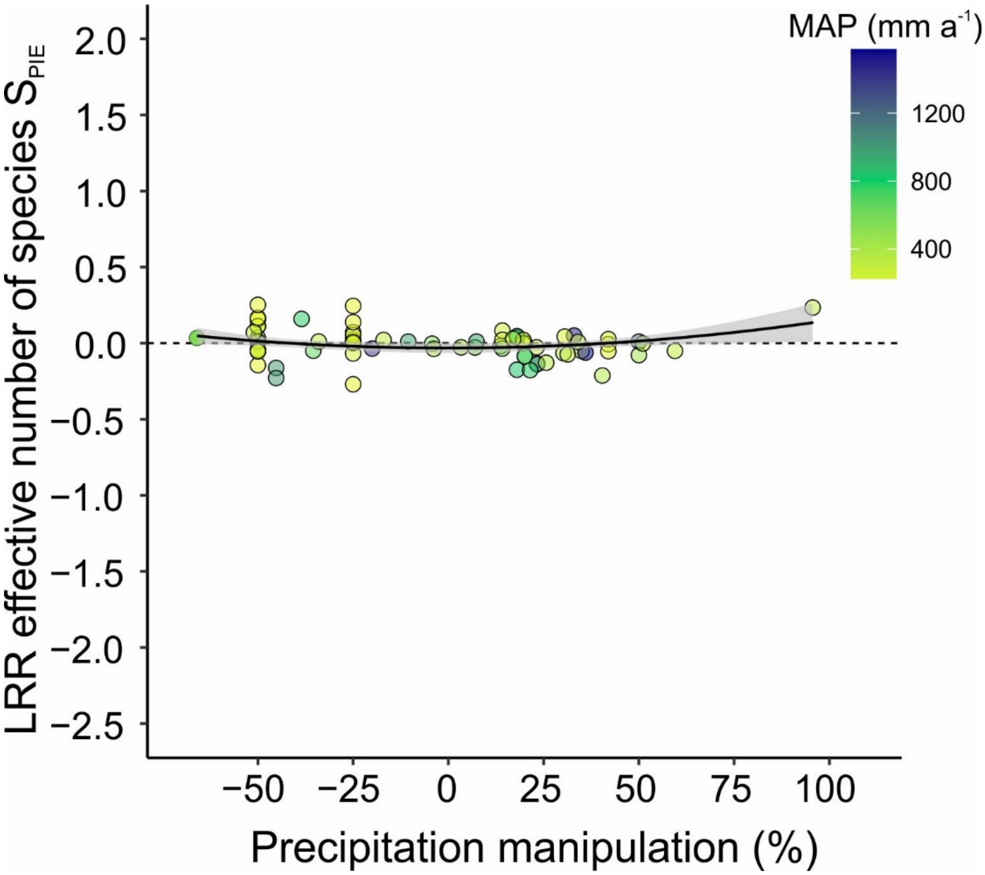

Supplementary Figure 7: Effect of the magnitude of precipitation manipulation on the log response ratio of the effective number of species S_{PIE} at the site scale. Data points represent log response ratios of original data and colors indicate the background mean annual precipitation (MAP). The non-linear regression (mean and 95% confidence intervals) is based on predicted values of the simplest mixed effect model including the nonlinear term of the magnitude of precipitation manipulation (Supplementary Table 4).

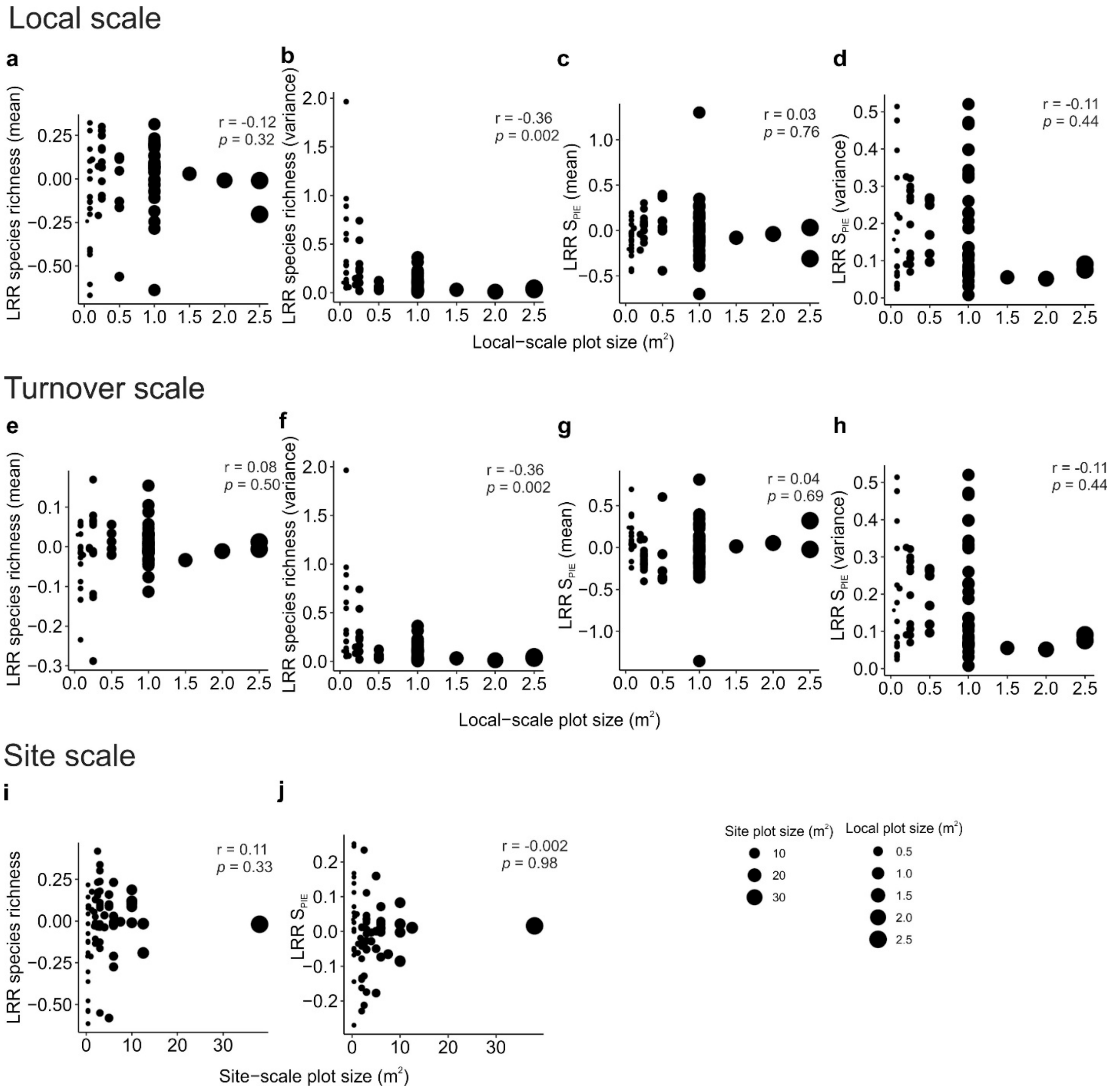

Supplementary Figure 8: Co-dependence between the mean and variance in the log response ratios of species richness (**a-b, e-f, i**) and effective number of species S_{PIE} (**c-d, g-h, j**) and plot size at the local (**a-d**), turnover (**e-h**) and site scale (**i-j**). Given are the correlation coefficient (r) from Pearson's product moment correlation for paired samples and *p*-values are derived from an associated t-test (two-sided). No adjustments were made for multiple comparisons.

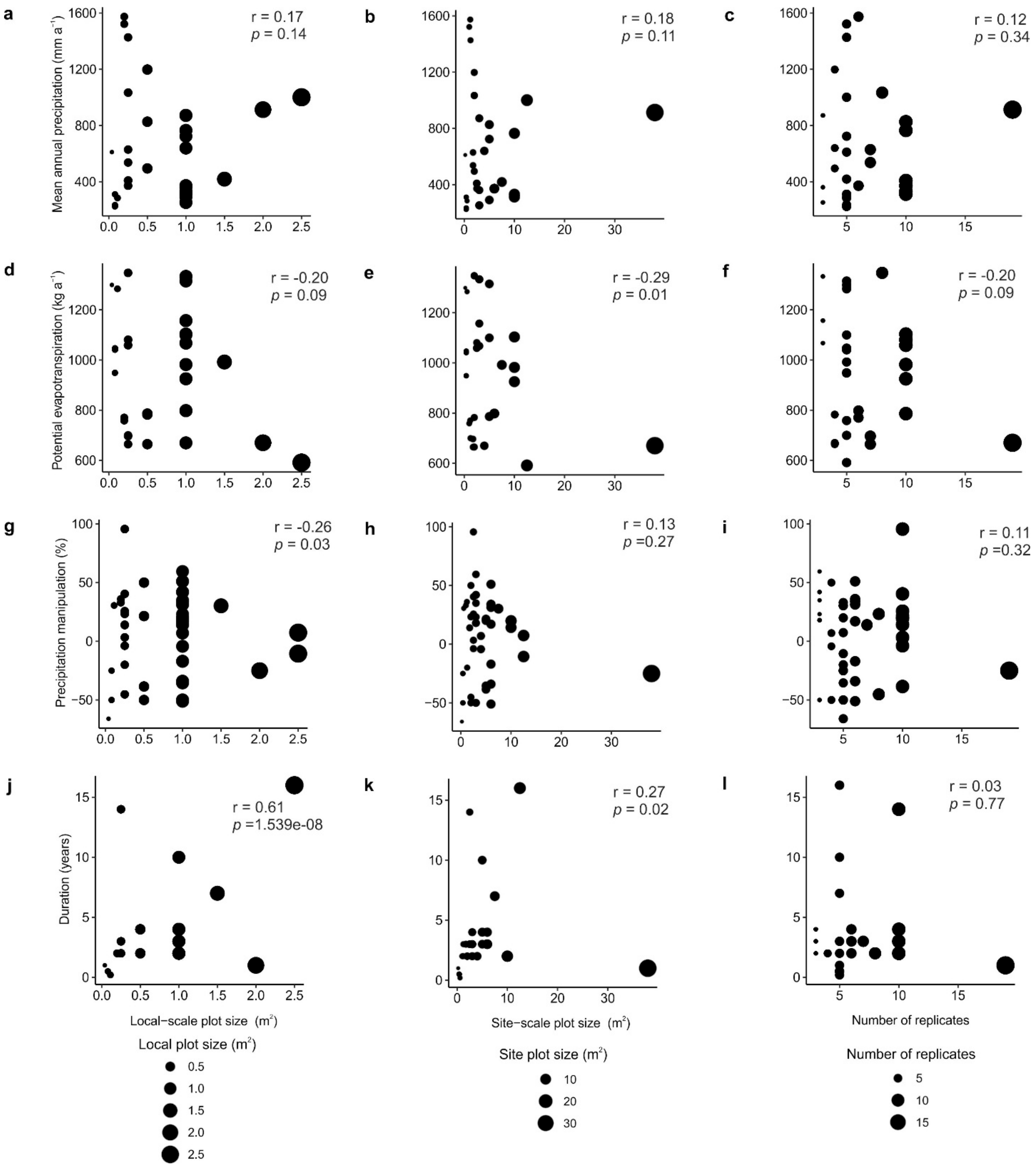

Supplementary Figure 9: Co-variation between plot size at the local-scale plot size (**a, d, g, j**), site-scale plot size (**b, e, h, k**) and number of replicates (**c, f, i, l**) and the moderators mean annual precipitation (**a-c**), potential evapotranspiration (**d-f**), magnitude of precipitation manipulation (**g-i**) and duration of the experiments **(j-l**). Given are the correlation coefficient (r) from Pearson's product moment correlation for paired samples and *p*-values are derived from an associated t-test (two-sided). No adjustments were made for multiple comparisons.

Supplementary References

1 Alon, M. & Sternberg, M. Effects of extreme drought on primary production, species composition and species diversity of a Mediterranean annual plant community. *Journal of Vegetation Science* 30, 1045-1061 (2019).

2 Báez, S., Collins, S. L., Pockman, W. T., Johnson, J. E. & Small, E. E. Effects of experimental rainfall manipulations on Chihuahuan Desert grassland and shrubland plant communities. *Oecologia* 172, 1117-1127 (2013).

3 Chimner, R., Welker, J., Morgan, J., LeCain, D. & Reeder, J. Experimental manipulations of winter snow and summer rain influence ecosystem carbon cycling in a mixed-grass prairie, Wyoming, USA. *Ecohydrology* 3, 284-293 (2010).

4 Collins, S. L. P., Wiliam; Fargione, Joe. Warming-El Nino-Nitrogen Deposition Experiment (WENNDEx): Net Primary Production Quadrat Data at the Sevilleta National Wildlife Refuge, New Mexico (2006 -present). doi:10.6073/pasta/564b856a2304244b48c2f744c1fbf619 (2011).

5 Eskelinen, A. & Harrison, S. P. Resource colimitation governs plant community responses to altered precipitation. *Proceedings of the National Academy of Sciences* 112, 13009-13014 (2015).

6 Fry, E. L. et al. Plant Functional Group Composition Modifies the Effects of Precipitation Change on Grassland Ecosystem Function. *Plos One* 8, e57027, doi:10.1371/journal.pone.0057027 (2013).

7 Gilgen, A. K. & Buchmann, N. Response of temperate grasslands at different altitudes to simulated summer drought differed but scaled with annual precipitation. *Biogeosciences* 6, 2525-2539, doi:10.5194/bg-6-2525-2009 (2009).

8 Grime, J. P. et al. Long-term resistance to simulated climate change in an infertile grassland. *Proceedings of the National Academy of Sciences* 105, 10028-10032, doi:10.1073/pnas.0711567105 (2008).

9 Hallett, L. M., Stein, C. & Suding, K. N. Functional diversity increases ecological stability in a grazed grassland. *Oecologia* 183, 831-840 (2017).

10 Harpole, W. S., Potts, D. L. & Suding, K. N. Ecosystem responses to water and nitrogen amendment in a California grassland. *Global Change Biology* 13, 2341-2348 (2007).

11 Holub, P., Fabšičová, M., Tůma, I., Záhora, J. & Fiala, K. Effects of artificially varying amounts of rainfall on two semi-natural grassland types. *Journal of Vegetation Science* 24, 518-529 (2013).

12 Kahmen, A., Perner, J. & Buchmann, N. Diversity-dependent productivity in semi-natural grasslands following climate perturbations. *Functional Ecology*, 594-601 (2005).

13 Lauenroth, W. SGS-LTER Effects of water and nitrogen additions on aboveground biomass in shortgrass ecosystems on the Central Plains Experimental Range, Nunn, Colorado, USA 1997-2011, ARS Study Number 143. doi:10.6073/pasta/55d7a673529b04d2bbd6fc7f6146703a (2014).

14 Ludewig, K. et al. Effects of reduced summer precipitation on productivity and forage quality of floodplain meadows at the Elbe and the Rhine River. *Plos One* 10, e0124140 (2015).

15 Mariotte, P., Vandenberghe, C., Kardol, P., Hagedorn, F. & Buttler, A. Subordinate plant species enhance community resistance against drought in semi-natural grasslands. *Journal of Ecology* 101, 763-773 (2013).

16 Miranda, J. d. D., Padilla, F., Lázaro, R. & Pugnaire, F. Do changes in rainfall patterns affect semiarid annual plant communities? *Journal of Vegetation Science* 20, 269-276 (2009).

17 Mueller, K. E. et al. Impacts of warming and elevated CO 2 on a semi-arid grassland are non-additive, shift with precipitation, and reverse over time. *Ecology Letters* 19, 956-966 (2016).

18 Prevéy, J. S. & Seastedt, T. R. Seasonality of precipitation interacts with exotic species to alter composition and phenology of a semi-arid grassland. Journal of Ecology 102, 1549-1561, doi:10.1111/1365-2745.12320 (2014).

19 Serafini, J., Grogan, P. & Aarssen, L. Summer precipitation limits plant species richness but not overall productivity in a temperate mesic old-field meadow. *Journal of Vegetation Science* 30, 832-844, doi:https://doi.org/10.1111/jvs.12783 (2019).

20 Smith, M. D. K., Alan. The Climate Extremes Experiment (CEE): Assessing ecosystem resistance and resilience to repeated climate extremes at Konza Prairie. doi:10.6073/pasta/57bbbfcf22783a52ad09d7239073a5de (2018).

21 Wilcox, K. R., von Fischer, J. C., Muscha, J. M., Petersen, M. K. & Knapp, A. K. Contrasting above- and belowground sensitivity of three Great Plains grasslands to altered rainfall regimes. *Global Change Biology* 21, 335-344 (2015).

22 Yang, H. et al. Community structure and composition in response to climate change in a temperate steppe. *Global Change Biology* 17, 452-465 (2011).

23 Zhong, M. et al. Asymmetric responses of plant community structure and composition to precipitation variabilities in a semi-arid steppe. *Oecologia 191*, 697-708 (2019)
