# Supplementary material for: Responses of plant diversity to precipitation change are strongest at local spatial scales and in drylands
Source: Nat Commun. 2021 May 3;12:2489. doi: 10.1038/s41467-021-22766-0 (PMC8093425; doi:10.1038/s41467-021-22766-0)
Supplement: Supplementary file 2 — Reporting Summary [file 41467_2021_22766_MOESM2_ESM.pdf]

nature research

Corresponding author(s): Lotte Korell

Last updated by author(s): Mar 1, 2021

Reporting Summary

Nature Research wishes to improve the reproducibility of the work that we publish. This form provides structure for consistency and transparency in reporting. For further information on Nature Research policies, see our Editorial Policies and the Editorial Policy Checklist.

Statistics

For all statistical analyses, confirm that the following items are present in the figure legend, table legend, main text, or Methods section.

n/a	Confirmed	
☐	☒	The exact sample size (*n*) for each experimental group/condition, given as a discrete number and unit of measurement
☐	☒	A statement on whether measurements were taken from distinct samples or whether the same sample was measured repeatedly
☐	☒	The statistical test(s) used AND whether they are one- or two-sided<br>*Only common tests should be described solely by name; describe more complex techniques in the Methods section.*
☐	☒	A description of all covariates tested
☐	☒	A description of any assumptions or corrections, such as tests of normality and adjustment for multiple comparisons
☐	☒	A full description of the statistical parameters including central tendency (e.g. means) or other basic estimates (e.g. regression coefficient) AND variation (e.g. standard deviation) or associated estimates of uncertainty (e.g. confidence intervals)
☐	☒	For null hypothesis testing, the test statistic (e.g. *F*, *t*, *r*) with confidence intervals, effect sizes, degrees of freedom and *P* value noted<br>*Give P values as exact values whenever suitable.*
☒	☐	For Bayesian analysis, information on the choice of priors and Markov chain Monte Carlo settings
☐	☒	For hierarchical and complex designs, identification of the appropriate level for tests and full reporting of outcomes
☐	☒	Estimates of effect sizes (e.g. Cohen's *d*, Pearson's *r*), indicating how they were calculated

Our web collection on statistics for biologists contains articles on many of the points above.

Software and code

Policy information about availability of computer code

Data collection	MS Excel and R (version 4.0.3.) were used in the data collection.
Data analysis	All data analyses were conducted using R statistical computing (version R 4.0.3.)<br>The associated analysis code is archived on github (https://github.com/lotte-korell/precipitation-change-experiments-.git).<br>Figures were produced in R but polished in CorelDraw (2018).

For manuscripts utilizing custom algorithms or software that are central to the research but not yet described in published literature, software must be made available to editors and reviewers. We strongly encourage code deposition in a community repository (e.g. GitHub). See the Nature Research guidelines for submitting code & software for further information.

Data

Policy information about availability of data

All manuscripts must include a data availability statement. This statement should provide the following information, where applicable:

- Accession codes, unique identifiers, or web links for publicly available datasets
- A list of figures that have associated raw data
- A description of any restrictions on data availability

The dataset that support the findings of this study are publicly available on Figshare (http://doi.org/10.6084/m9.figshare.14061260) and the climate data are publicly available on the Chelsa database (http://doi.org/10.5061/dryad.kd1d4).

Field-specific reporting

Please select the one below that is the best fit for your research. If you are not sure, read the appropriate sections before making your selection.

☐ Life sciences ☐ Behavioural & social sciences ☒ Ecological, evolutionary & environmental sciences

For a reference copy of the document with all sections, see nature.com/documents/nr-reporting-summary-flat.pdf

Ecological, evolutionary & environmental sciences study design

All studies must disclose on these points even when the disclosure is negative.

Study description	This synthesis compiled data from 72 precipitation manipulation field experiments originating from 23 studies that investigated the effects of altered precipitation on plant biodiversity. The included experiments had different designs (i.e. completely randomized, randomized block, split-plot design) and the precipitation manipulations were increase in precipitation (i.e. watering) and/or decrease in precipitation (i.e. rainout shelters). For each experiment we extracted the magnitude of precipitation change and tested wether the effects on biodiversity depended on spatial scale and background climatic conditions.
Research sample	Each study on how precipitation affected the plant community diversity and composition contained at least three replicates of control and treatment plots per site, which resulted in 462 control-treatment comparisons at the local scale (plot level) and 72 control-treatment comparisons at the site level. In the analysis a hierarchical random term was specified to control for the non-independence between experiments at the different sites and studies.
Sampling strategy	As this was a meta-analysis of precipitation experiments, studies differed widely in their sampling design. For example, there are different measures of plant species abundance (% cover, bm, point intercept) but by using relative rather than absolute abundances for the analysis and by using log response ratios we were able to compare effect sizes across studies with different sampling design and method. Furthermore, standardization by sample size was done if possible.
Data collection	As this is a meta-analysis of 23 studies many people were involved in the original data collection. The data compilation which was lead by the correspoding author was based on a systematic literature search, followed by data request (see Fig. S5 for details). Data were compiled in MS excel and handled in R.
Timing and spatial scale	As this is a meta-analysis the time period ranged from datasets collected from the 1994 - 2016. Most of the studies were conducted in North America and Europe. Spatial scale and sampling effort varied considerably between studies, but we carefully checked for co-variation between sample size/effect size and if possible we controled for sampling effort so that standardization could take place.
Data exclusions	No data was excluded from this study.
Reproducibility	Details about the literature search for this synthesis and the selection criteria for study inclusion can be found in the Methods section of the paper. Furthermore, information on the number of replicate can be found in Table S1 and a sensitivity analysis of how the effect size depended on sample size is provided in Fig. S9
Randomization	Based on our systematic literature search, we have used all data that was publicly available or provided by the authors to carry out a synthesis of existing knowledge. Because this sample is biased, we addressed such bias in the analyses (e.g how mean and variance of effect sizes depend on the plot size and sample size, Fig S8 & S9) and we addressed the role of geographic biases in the discussion of the article.
Blinding	Blinding is not used in the meta-analysis of already published data.

Did the study involve field work? ☐ Yes ☒ No

Reporting for specific materials, systems and methods

We require information from authors about some types of materials, experimental systems and methods used in many studies. Here, indicate whether each material, system or method listed is relevant to your study. If you are not sure if a list item applies to your research, read the appropriate section before selecting a response.

Materials & experimental systems

n/a	Involved in the study	
☒	☐	Antibodies
☒	☐	Eukaryotic cell lines
☒	☐	Palaeontology and archaeology
☒	☐	Animals and other organisms
☒	☐	Human research participants
☒	☐	Clinical data
☒	☐	Dual use research of concern

Methods

n/a	Involved in the study	
☒	☐	ChIP-seq
☒	☐	Flow cytometry
☒	☐	MRI-based neuroimaging
